# An Innovative Method for the Recycling of Waste Carbohydrate-Based Flours

**DOI:** 10.3390/polym12061414

**Published:** 2020-06-24

**Authors:** Carola Esposito Corcione, Raffaella Striani, Francesca Ferrari, Paolo Visconti, Daniela Rizzo, Antonio Greco

**Affiliations:** 1 Department of Engineering for Innovation, University of Salento, 73100 Lecce, Italy; raffaella.striani@unisalento.it (R.S.); francesca.ferrari@unisalento.it (F.F.); paolo.visconti@unisalento.it (P.V.); antonio.greco@unisalento.it (A.G.); 2 Department of Cultural Heritage, University of Salento, 73100 Lecce, Italy; daniela.rizzo@unisalento.it

**Keywords:** thermoplastic bio-film, waste flour, maize starch, mass transport properties, durability, solubility in water

## Abstract

This work represents an innovative study that, for the first time, explores the possibility to use waste flours to produce thermoplastic polymeric bio-films. To the best of our knowledge, this is the first time that waste flours, derived from bakeries, pizzerias or pasta factories, have been proposed for the production of bio-polymers, as a replacement of neat starch. To this aim, durum waste flour derived from a pasta factory, soft waste flour derived from pizzerias and neat maize starch used as control material were firstly analyzed from dimensional, morphological and chemical points of view. Afterwards, waste flour films were produced by the addition of a nature-based plasticizer, glycerol. Mechanical characterization of the plasticized thermoplastic films, produced by compression molding, evidenced low performances, even in the case of the neat maize starch. In order to improve the mechanical properties, the possibility to include polylactic acid and cardanol-based plasticizer was also investigated. Mass transport properties of all the produced bio-films were investigated by measuring their water vapor permeability and hygroscopic absorption. The durability properties of the bio-films were assessed by accelerated ageing tests, while the bio-degradability of the waste-based films was evaluated by measuring the solubility and the degradation in water. The physicochemical analyses of the novel bio-films evidenced good mechanical properties; specifically, the waste-based films showed a lower hygroscopic absorption and water solubility than those of the blends containing neat starch.

## 1. Introduction

For more than a century, starch has been the most widely used natural polysaccharide for the production of polymeric bio-films because of its wide availability in agriculture, biodegradability and cheapness. It is considered one of the most abundant renewable materials, but, if compared to the synthetic polymers, it presents two great disadvantages: a high hygroscopicity, due to the presence of hydroxyl groups [1], and a narrow processing window, due to fast degradation. In fact, a simultaneous pyrolysis with the melting of the crystalline structures could be possible [2,3]. The processability of the starch, hence, is feasible only by destroying the crystalline structure. This phenomenon occurs by heating starch in conditions of high pressure and shear, with a proper amount of plasticizer (such as water or polyols) [4,5] that decreases the melting temperature. Furthermore, it must be considered that, during the cooling, the retrogradation of the starch could occur. In fact, a reversible process of rearrangement of the amylose and amylopectin chains of the gelatinized starch takes place, and the amylopectin chains tend to return by returning to a similar configuration as their its initial one, with the consequent loss of a part of the water that had been incorporated into the structure during the gelatinization phase. For this reason, the polymer chains of the thermoplastic starch can recrystallize in several crystalline structures for the separation and migration of the plasticizer by causing a variation of the mechanical properties [2,5,6]. Successful procedures for the production of thermoplastic starch film by means of extrusion and compression molding were developed by Giuri et al. [7] and Stasi et al. [8], respectively. The method proposed by Giuri et al. [7] involved a controlled, easy and cheap processing procedure by using an extrusion technique and by adding 50 wt % glycerol to the maize native starch. Nevertheless, mechanical properties of the starch films were lower than those of the synthetic thermoplastic polymer films, and the hygroscopic behavior of the starch thermoplastic films was still high, irrespective of their composition [7]. Stasi et al. [8] used compression molding to process native starch, in presence or absence of carbon-based waste ashes. They found that the presence of ashes decreases the biodegradability of the starch by 70%, independently of the filler amount. In addition, the tensile tests showed a relevant stiffening effect of the filler, with an increase of about 20% in tensile modulus. Besides, the moisture absorption capacity of the thermoplastic starch films decreased by about 30% thanks to the presence of the waste carbon ashes [8].

Several other attempts reported in the literature have been made to increase the mechanical properties of thermoplastic starch films by blending the starch with polylactic acid (PLA) [9,10,11,12,13,14]. PLA is a promising thermoplastic polymer derived from agricultural resources through bio-conversion and polymerization of the starch. Its stiffness, tensile strength and gas permeability are comparable to those of synthetic polymers from fossil fuels [9,10,12]. Wang et al. [15] produced thermoplastic dry starch (DTPS)/PLA blends using maleic anhydride (MA) as the compatibilizer, evidencing that MA improved the interaction between DTPS and PLA and, at the same time, the blend became more thermally stable. Li and Huneault [16] investigated the use of glycerol and sorbitol as thermoplastic starch (TPS) plasticizers in TPS/PLA blends. They found that the sorbitol-plasticized TPS phase can be more finely dispersed and more uniformly distributed in the PLA matrix even in the absence of any compatibilization. The sorbitol-plasticized TPS/PLA blends exhibited much higher tensile strength and modulus. They were also shown to be less volatile in thermogravimetric measurements. However, despite the interesting results reported in the literature on both native starch and starch-based blends [7,9,10,11,12,13,14,15,16,17,18], an important issue remains unresolved: in fact, all the developed approaches use a native polysaccharide, which could instead be used as a nutritional resource for people in the developing countries. On the other hand, one of the major challenges in management and disposal is the excessive waste stream deriving from the different sectors of the current linear economic model. Municipal waste accounts for only about 10% of the total waste generated. Municipal solid waste consists of waste collected by or on behalf of municipal authorities and disposed through the waste management system. Several studies were carried out by the authors in order to valorize the organic fraction of municipal solid waste [19,20,21,22,23].

Waste flours, coming from bakeries, pasta factories and food-producing industries, are an example of a selected municipal solid waste, and they are now often widely used in animal feed, as compostable products or as heating fuel. Flour is made from a starchy food material that contains a high proportion of complex carbohydrates, also known as polysaccharides. Therefore, flour, instead of pristine starch, can be used to produce edible and biodegradable films. Muangrat and Nuankham [24] already explored the possibility to produce flour-based films by using waste flour from the rice-noodle-producing industry. They also used the best produced bio-film as a natural packaging material, thus demonstrating for the first time that waste flour films could be a promising alternative material to reduce the use of non-biodegradable synthetic polymer films in food applications. However, the drawbacks related to the use of waste flours are mainly due to both the presence of low mechanical properties and the high susceptibility to moisture or water.

Downstream of a previous study [25], the present work aims to develop novel techniques for enhancing the intrinsic and economic value of all kind of waste flours derived from bakeries, pizzerias or pasta factories. In fact, the main objective of the conducted research work is to develop biotechnological and green chemistry pathways for the production of high-added-value biopolymers with low environmental impact, hence also allowing the reuse of the waste flours and the creation of an added-value reusable bio-product.

## 2. Experimental

### 2.1. Materials

The commercial maize starch (labeled as MS) was purchased from Merck-Sigma Aldrich company, (Milan, Italy, Product No. 9005-25-8) and used in this work as a control material for comparison purposes. The commercial maize starch consists of 27% amylose (linear glucose polymer) and 73% amylopectin (highly branched glucose polymer) [26].

The durum wheat semolina flour (labeled as DF), generally consisting of about 23% amylose and 77% amylopectin [27], and soft wheat semolina flour (labeled as SF), commonly consisting of about 22% amylose and 78% amylopectin [28], were purchased at the supermarket and were characterized for comparison purposes. The durum waste flour (labeled as DWF) was obtained from the processing waste of the Benedetto Cavalieri S.r.l. pasta factory (Lecce, Italy) and then dried at 105 °C for 24 h in order to reduce the moisture level to zero. Instead, the soft waste flour (labeled as SWF) was obtained from the processing waste of pizzerias; it was previously cleaned of the impurities and was finally dried at 105 °C for 24 h.

All the flours were stored in an oven at 75 °C for 24 h before use. The maize starch and the waste flours were plasticized with glycerol (labeled as G) supplied by Cruciani Crual S.r.l. (Rome, Italy).

The polylactic acid used in this work is the polyester Ingeo Biopolymer 2003D, supplied by NatureWorks (Minnetonka, MN, US), characterized by a density of 1.24 g cm^−3^ and a melt flow rate of 6 g/600 s at a temperature of 210 °C. According to the producer’s technical data sheet, the polymer is mainly composed of L-isomer, with the D-isomer content lower than 4%. PLA was plasticized with cardanol (labeled as C) purchased from Oltremare company (Bologna, Italy) and characterized by a purity of 95%.

#### Preparation of the Bio-Films

For the present research work, three kinds of bio-based thermoplastic blends were developed. The first type was labeled *Type 0* and corresponds to a bio-based thermoplastic polymer consisting of starch or waste flours plasticized with glycerol; the second kind was named *Type 1* and consists of a bio-based thermoplastic polymer blend, composed of starch or waste flours and PLA, plasticized with glycerol; finally, the *Type 2* bio-based thermoplastic polymer blend was produced by mixing starch or waste flours and PLA, plasticized with glycerol and cardanol (with a 50/50 ratio of powder glycerol to PLA cardanol: 50/50).

All the produced blends and their compositions are reported in Table 1. Specifically, taking into account the acronyms of used materials as reported in the caption of Table 1, *Type 0* blends are labeled as “X-Number-G”, where X is the used flour, G is the glycerol and Number is the weight amount of G with respect to X. Instead, *Type 1* blends are labeled as “X-Number-G_PLA (ratio)”, where the ratio represents the weight amount of “X-Number-G” with respect to the PLA. Finally, for *Type 2* blends, labeled in Table 1 as “X-Number-G_PLA-Number2-C (ratio)”, Number 2 is the weight amount of “C” in the blend.

Bio-films were produced by a preliminary mixing (by employing a HAAKE Rheomex 600/610 mixer, manufactured by Thermo Fisher Scientific, Waltham, MA, USA) with a rotor speed of 100 rpm. Then, the mixed blends were melted in an oven with a holding time of 1 min at the processing temperature and finally molded in square shapes (with dimensions of 150 mm × 150 mm × 0.5 mm) at the pressure of 50 bar by means of compression molding (specifically, by using a vertical press, model P7/91, manufactured by DGTS Srl, Veduggio (MB), Italy).

Following the results obtained in a previous work [7], the films for each blend were produced in different ways. Specifically, for the *Type 0* bio-films, the powder (MS, DWF or SWF) and glycerol were mixed in proper amounts for 15 min at 130 °C with a rotor speed of 100 rpm (with different weight percentages, as previously detailed); after, the blends were melted in an oven at 130 °C before being pressed at a pressure of 50 bar. For the production of *Type 1* bio-films, the same powders with glycerol were mixed as previously reported; then, the PLA was added by increasing the processing temperature up to 150 °C, in order to allow the melting of the PLA, and then mixed for 5 min. Finally, the blends were pressed at a pressure of 50 bar. The *Type 2* blends were produced by mixing the selected flour with glycerol according to the previous procedure; then, the PLA and a proper amount of cardanol were added and mixed for 5 min, after increasing the temperature to 150 °C. Finally, the blends were processed like the *Type 1* ones by pressing them at a pressure of 50 bar.

### 2.2. Methods

#### 2.2.1. Flour Powder Characterization

A preliminary characterization of the starch and waste flours was carried out in order to study the morphology, the degree of crystallinity and the molecular groups. The particles’ average diameter was calculated by means of multi-angle light scattering (MALS) with a CILAS 1190 particle size analyzer (manufactured by CPS US Inc., Madison, WI, USA). The degree of crystallinity was assessed from the spectra acquired by employing an Ultima IV X-ray diffraction (XRD) system (manufactured by Rigaku Company, Austin, TX, USA), according to the formula proposed by Hermans and Weidinger [29]:

(1) $$\%Cristallinity=\frac{Q_{st}-Q_{am}}{Q_{st}}*100$$

where *Q_st_* and *Q_am_* are the areas, as determined by the XRD spectra, related to the semicrystalline and amorphous patterns, respectively.

In order to establish the glycerol content required to plasticize the maize starch and the waste flours, dynamic rheological scans in the range of 30 to 200 °C were carried out by means of a Rheometrics Ares rheometer (manufactured by TA Instruments, New Castle, DE, USA) for evaluating the slope of viscosity as a function of the temperature. Rheological measurements were also employed to study the proper temperature for processing the powder samples and for realizing bio-films.

#### 2.2.2. Bio-Film Characterization

The mechanical properties of the bio-films from Table 1 were determined by means of tensile tests according to the ASTM D882-97 standard test method [30]. Five replications were performed for each film (with dimensions of 120 mm × 10 mm × 0.5–0.7 mm) by using a dynamometer (model Lloyd LR5 K, manufactured by Lloyd Instruments Ltd., Bognor Regis, UK) equipped with a load cell of 5 kN and displacement speed of 5 mm/min.

Dynamic mechanical analysis (DMA) was performed on plasticized bio-film samples by using a strain-controlled Rheometrics Ares rheometer, with a torsion geometry, by increasing the temperature from 30 to 120 °C with a heating rate of 2 °C/min.

The differential scanning calorimetry (DSC) method was employed for measuring the glass transition temperature, Tg, of the bio-films (by employing the DSC 622 Instrument, manufactured by Mettler Toledo, Columbus, OH, USA). Dynamic scans were performed in a nitrogen atmosphere from 25 to 230 °C, with a rate of increase of 10 °C/min; at least three tests were done on each sample.

The hygroscopic absorption of the bio-films reported in Table 1 was assessed under controlled environmental conditions, specifically with a relative humidity (RH) equal to 75% at 23 °C in a climatic chamber (model BINDER KBF 115, Binder, Tuttlingen, Germania). The moisture absorption was measured by weighing the bio-films at regular time intervals up to saturation. The imbibition coefficient, α, was then calculated according to the following equation:

(2) $$\alpha \left(\%\right)=\frac{W_{t}-W_{0}}{W_{0}}*100$$

where *W_t_* is the mass at time *t* and *W_0_* is the initial mass.

The water vapor permeability was measured for all the bio-films by means of the Multiperm ExtraSolution instrument (manufactured by PermTech Srl, Pieve Fosciana, Italy). The tests were carried out on a surface area equal to 2 cm^2^ at a temperature of 23 °C and RH 90 % ± 1.5%.

In order to assess the durability of the bio-films, they were subjected to one cycle of ultraviolet (UV) exposure for 8 h (irradiance equal to 0.76 W/m^2^ and temperature of 60 °C) and condensation exposure for 4 h (at the temperature of 50 °C), according to the ISO 4892-3 method cycle K in a QUV (Q Ultraviolet) chamber (manufactured by Q-Lab, Saarbrucken, Germany) equipped with UV-A lamps (λ equal to 340 nm).

The effects of the QUV ageing were evaluated by means of the total color variation (by using Konica Minolta CR-410 colorimeter, manufactured by Konica Minolta Sensing, Tokyo, Japan) under conditions of total reflectance and double channel mode, using a Xenon lamp light source. The color changes were evaluated by the L* a* b* system [31] and expressed as ΔE, defined according to the following equation:

(3) $$\Delta E=\left(\sqrt{\Delta L^{*2}+\Delta a^{*2}+\Delta b^{*2}\;}\right)$$

The QUV ageing effects have also been evaluated by means of the tensile test [30] in order to compare the mechanical properties before and after the QUV exposure.

The solubility in water was assessed by drying of the samples at 105 °C for 24 h, weighing the dried samples (W_0_), and then immersing them in distilled water at different temperatures, i.e., 25 and 50 °C, for 24 h. This was followed by drying the insoluble part of the samples at 105 °C for 24 h and then weighing (W_in_) the samples. The solubility in water of each bio-film was calculated according to the following equation:

(4) $$Solubility\;\left(\%\right)=\frac{W_{0}-W_{in}}{W_{0}}*100$$

where W_0_ is the dry weight of the samples and W_in_ is the weight of the insoluble part of the bio-film.

## 3. Results

### 3.1. Characterization of Flour Powders

The initial aim of the characterization of the raw materials was to study the morphological, structural and physicochemical properties of the waste flours. For this reason, granulometric and diffractometric analysis were performed on both neat and waste flours. In particular, neat MS was used as a control material due to its widespread employment for the production of thermoplastic films; both waste flours, namely DWF and SWF, were compared to the corresponding produced flours, namely DF and SF. In Figure S1, the granulometric distribution curves of all the powders are reported, while the diameters of the particles related to the same powders are collected in Table S1.

The processing of flours changed the structure by reducing the particles’ diameter for both durum and soft wheat flours. In particular, by comparing the neat with the corresponding waste flour, at 90% of the cumulative value, the grain size decreased by about 67% for DWF and about 47% for SWF.

The characteristic diffractometric peaks of maize starch (see the inset of Figure S2) are also visible in neat and waste flour spectra, confirming the literature data [6] about the carbohydrate nature of the samples. The XRD spectra reported in Figure S2 confirm an evident variation of the crystallinity for the DWF with respect to as produced DF. Instead, the SWF seems to have undergone no changes in the degree of crystallinity. In fact, by calculating the degree of crystallinity of all the flour samples, according to the Equation (1), a noticeable increase of crystallinity was marked just for DWF with respect to the DF, as reported in Table S2.

**Figure 1:**
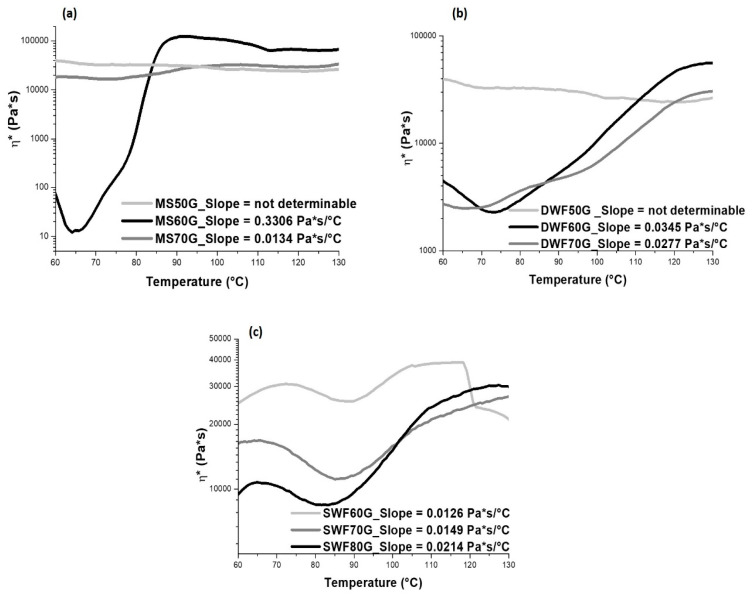
Rheological analysis of maize starch (MS) (**a**), durum waste flour (DWF) (**b**) and soft waste flour (SWF) (**c**) with different glycerol contents.

In order to define the amount of plasticizer for starch and the waste flours, dynamical rheological scans were performed at different plasticizer contents. The results are reported in Figure 1.

From the rheological curves reported in Figure 1, the increase of the viscosity, due to absorption of the glycerol from the starch particles and consequent swelling, starts at about 70–75 °C for both MS60G and DWF60G blends (Figure 1a,b, respectively). Instead, for SWF, the swelling process occurs at a higher temperature (over 85 °C). The optimal glycerol amount for each powder was chosen as the one corresponding to the fastest plasticizer absorption, which corresponds to faster swelling and therefore to the highest slope in the viscosity curves. According to the results reported in the legend of Figure 1, this corresponds to 60% glycerol in MS and DWF and 80% glycerol in SWF.

### 3.2. Characterization of the Bio-Films

In Figure S3, some images of the produced bio-films are reported for DWF60G (*Type 0*), DWF60G_PLA (*Type 1*) and DWF60G_PLA30C (*Type 2*) samples. In order to assess the performances of the bio-films, the mechanical, thermal and transport properties of all the bio-films were evaluated.

Firstly, the mechanical properties of the waste-flour-based films (DWF60G and SWF80G) were measured and compared to those of the starch-based film (MS60G). In Table 2, the tensile properties of all bio-based films are reported; these experimental data evidence a decrease of the tensile properties of both the waste-flour-based films with respect to the control material (namely neat maize starch). In particular, both waste flour films have comparable values for strain at break, but these are about 75% lower than those of maize-based films. By comparing the ratio of tensile strength, there are noticeable decreases of about 20% and 45% for DWF60G and SWF80G, respectively, in comparison to MS60G.

In order to improve the tensile properties of the waste-based films (*Type 0* ones), bio-thermoplastic polymer blends have been produced with the addition of PLA (*Type 1* bio-films), according to the composition given in Table 1. As expected, for all the *Type 1* bio-films, the introduction of PLA brought an increase of tensile strength and modulus with respect to the MS60G film (Table 2). On the other hand, the presence of PLA caused a decrease of the strain at break. For this reason, in order to find a compromise between a good tensile strength and an acceptable strain at break, the *Type 1* blends with the 50/50 ratio were also selected for the production of the *Type 2* blends, according to the composition reported in Table 1. The properties reported in Table 2 show that the plasticization by cardanol allowed the strain at break of bio-films to be increased, although this was accompanied by decreases in the tensile strength and modulus. In particular, the blends of *Type 2* show better mechanical performances compared to MS60G.

A comparison of the mechanical properties of the DWF60G_PLA30C bio-films with those of the films developed from noodle rice by Muangrat and Nuankham [24], shows a comparable tensile strength and much better strain at the break (with an increase of about 60%). On the other hand, the mechanical properties of SWF80G_PLA30C bio-films show a comparable strain at break and lower strength compared to the films produced by Muangrat and Nuankham [24].

The present mechanical results could be also compared with those obtained by Mendes et al. [17], who developed biodegradable thermoplastic polymer blends based on native corn starch or chitosan. In particular, Mendes et al. [17] found better performances for chitosan-based films; however, they had slightly lower tensile strength and deformation (1.5 MPa and 108%, respectively) than those of DWF60G_PLA30C bio-films. Furthermore, it must be considered that the thermoplastic films produced by Mendes et al. [17] were derived from neat raw materials, whereas the bio-films developed in this work were derived from waste flours.

The glass transition onset, T_onset_, calculated as the onset temperature of storage shear modulus, decreases [32], whereas the Tg values, calculated as the inflection point by the DSC methodology, are reported in Table 3. The DSC curves, from which the Tg values shown in Table 3 have been obtained, are presented in Figure S4.

From the values reported in Table 3, it is possible to note the same behavior for all types of waste-flour-based films (namely *Types 0*, *1* and *2*, as reported in Table 3). Specifically, when the PLA is added (*Type 1* films), the Tg is reduced, due to the lower glass transition of PLA compared to *Type 0* blends [33,34]. On the other hand, when cardanol is added, its plasticizing effect towards PLA involves a significant further decrease of Tg [33]. For the maize-starch-based control, the Tg values are about 10 °C lower than those of DWF and SWF. The trend of the glass transition temperature recorded for each type of bio-films is confirmed by the DSC tests as well.

**Figure 2:**
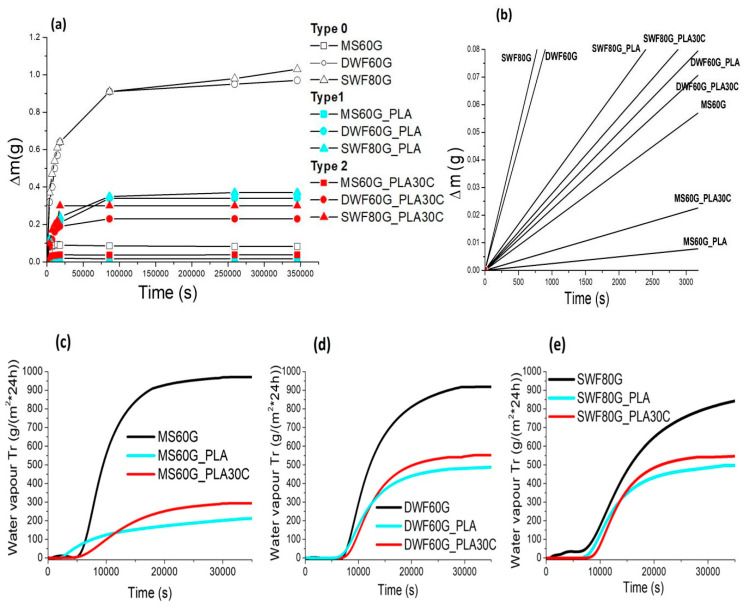
Hygroscopic absorption curves as a function of time (**a**) and a time magnification related to the first linear section (**b**); water vapor permeability curves of MS-based bio-films (**c**), DWF-based bio-films (**d**) and SWF-based bio-films (**e**).

In Figure 2 the results of the hygroscopic absorption (at the temperature of 23 °C and RH equal to 75%) and water vapor permeability (at 23 °C and RH equal to 90%) are reported for each realized bio-film typology. The imbibition coefficient, reported in Table 4, has been calculated according to the Equation (2).

By considering the values in Table 4, it is firstly noticeable that the imbibition coefficient and the absorption rate of MS60G confirm the previously reported α values of the starch-based films (around 13%) [7]. On the other hand, α values and absorption rate of both waste-flour-based *Type 0* films are higher than those of the control MS60G. According to XRD analysis (Table S2), it is noticeable that the degree of crystallinity of the maize starch exceeds that of the waste flours, which justifies the marked hygroscopic properties of the latter.

The addition of PLA (*Type 1* bio-films) involves, for all the different powders, a significant reduction of the imbibition coefficient and the slope. In fact, for the *Type 1* bio-films, the imbibition coefficient decreases by 60% for DWF60G_PLA and 56% for both the MS60G_PLA and SWF80G_PLA bio-films. This behavior can be explained by accounting for the higher moisture absorption of each type of flour (both starch and waste flours) characterized by an abundance of hydroxyl groups which interact with the water molecules [35,36,37] compared to PLA. This result is also confirmed by the water vapor permeability analysis; in fact, as evidenced by the curves reported in Figure 2c–e and by the data in Table 4, all the *Type 0* bio-films (black lines) show the highest water vapor permeability values, around 900 g/m^2^ per day. The slopes, calculated in the first linear section of the curves, also confirm that the water vapor transmission rate is greater for *Type 0* bio-films with respect to the corresponding *Type 1* and *Type 2* films. Analogously to the imbibition results, the water vapor permeability data also evidence a reduction when the PLA is added in *Type 1* bio-films. In particular, a decrease in water vapor transmission of about 78% is noticeable for starch-based bio-films, and decreases of 47% and 35% are noticeable for DWF and SFW based bio-films, respectively. On the other hand, the plasticization of PLA in *Type 2* bio-films does not induce any evident changes in terms of both imbibition coefficient and water vapor permeability with respect to the *Type 1* bio-films, but just a slight increase.

By comparing the moisture absorption data recorded by Stasi et al. [8], who developed compression-molded composite films of thermoplastic carbon-based ashes/maize starch, it is noticeable that the imbibition coefficient values determined for *Type 1* and *Type 2* bio-films are lower than those in presence of carbon ashes (with a percentage reduction in the interval of 13%–20%), even though the ashes do not contribute to the water absorption of the starch films [8].

#### Artificial Ageing

A further investigation aimed to assess the durability of the developed bio-films by means of artificial ageing tests. For this reason, all the bio-films have been subjected to testing by QUV exposure and in terms of degradation in water.

**Figure 3:**
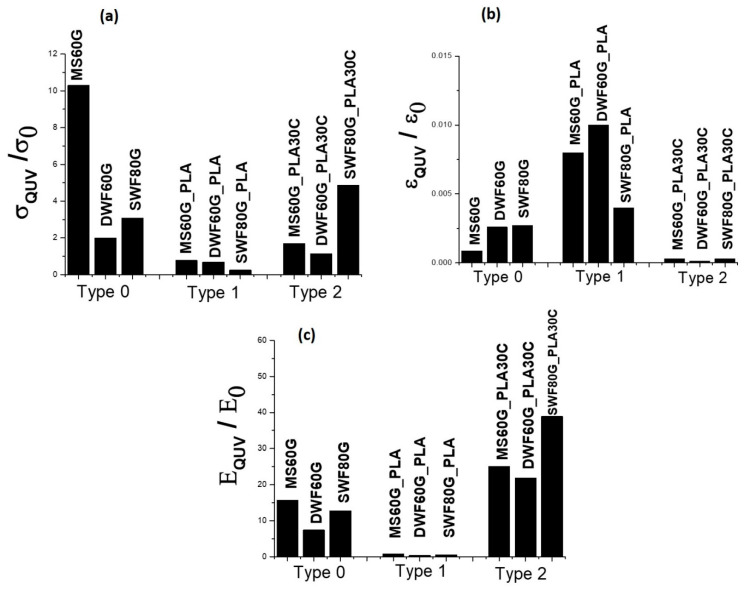
Ratios of tensile strength (**a**), deformation (**b**) and elastic modulus (**c**) of maize starch (MS)-based, durum waste flour (DWF)-based and soft waste flour (SWF)-based bio-films before and after QUV ageing.

**Figure 4:**
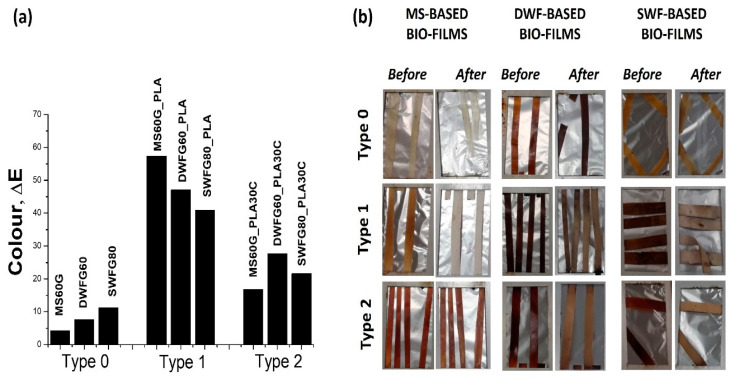
Total color variation (ΔE) (**a**) and images before and after the QUV ageing (**b**) of the maize starch (MS)-based, durum waste flour (DWF)-based and soft waste flour (SWF)-based prepared bio-films.

In order to evaluate the QUV ageing effect, color measurements and tensile tests were carried out before and after the UV/condensation cycle. The ratios of mechanical properties and color variation before and after the QUV ageing are shown in Figure 3 and Figure 4, respectively.

For all the *Type 0* samples, QUV involves a significant improvement of strength and stiffness, despite a decrease of the strain at break, which highlights the significant effect of QUV towards embrittlement of the material. A similar effect can be observed for *Type 2* samples; in this case, the increase in modulus and decrease in strain at break decrease are even more evident, highlighting that the addition of glycerol and cardanol involves a severe degradation of the films during QUV exposure. *Type 1* samples, namely those with the lowest overall plasticizer content (glycerol), are the least sensitive to QUV, since only slight decreases of the strength and stiffness are detected. However, these samples allow the initial strain at break to better retained; however, this value is still much lower than that before the UV exposure. On the other hand, the color variation, expressed as ΔE, that indicates the total color difference compared to before ageing, as reported in the graph of Figure 4a, shows very high aesthetical alteration for all the *Type 1* bio-films, recording ΔE values in the 40–60 range. Giuri et al. [7] recorded ΔE values in the 45–55 range for the extruded thermoplastic maize-starch-based films, evidencing an increase in yellowing during the UV-lamp exposure time.

All the data related to the color analysis are reported in the following Table 5. The chromaticity coordinates of the bio-films in the CIE (Commission Internationale de l’Eclairage) color space, where L* is the lightness, a* is the green–red color range and b* is the blue–yellow color range, evidence that the photodegradation induced by the QUV ageing mostly affects the blends with PLA (namely *Type 1* and *Type 2* bio-films), for which the highest color difference values were recorded (as shown in Figure 4b). In particular, the UV radiation caused a noticeable lightness variation for MS60G_PLA and DWF60G_PLA bio-films and for all *Type 2* samples, whereas, for the bio-film SWF80G_PLA, a more evident deviation was assessed in the a* and b* coordinates.

**Figure 5:**
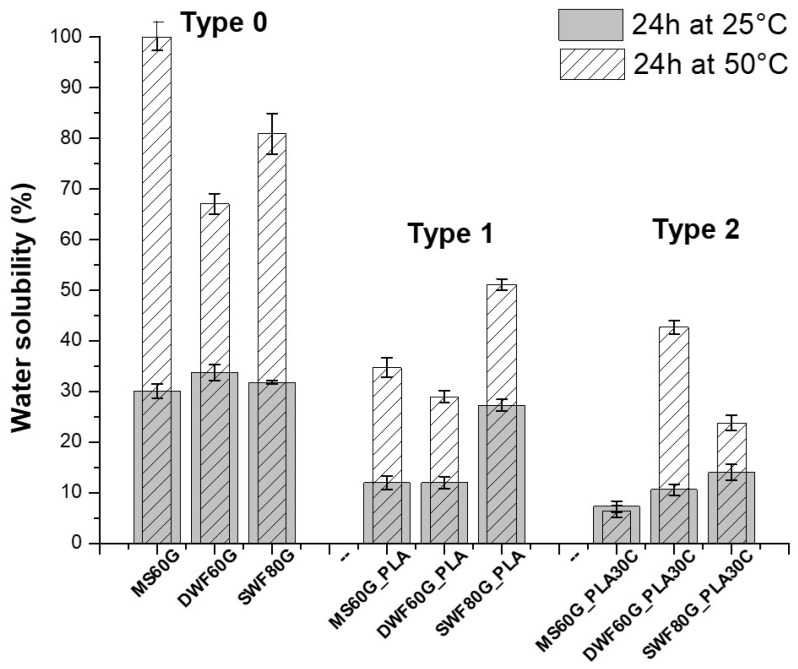
Water solubility at 25 and 50 °C for 24 h of maize starch (MS)-based, durum waste flour (DWF)-based and soft waste flour (SWF)-based bio-films.

The solubility of bio-films assessed by immersion in water at different temperatures, i.e., 25 and 50 °C, was calculated within the first 24 h (Figure 5).

As evidenced in Figure 5, the water solubility at 50 °C calculated within the first 24 h is very high for *Type 0* bio-films and less than 50% for *Type 1* and *Type 2* films. Instead, in non-severe conditions (at 25 °C), all the bio-films show a very low solubility; in particular, for both MS60G_PLA and DWF60G_PLA *Type 1* samples and for all *Type 2* bio-films, a solubility in water of less than 15% has been recorded. If compared to the literature data [24], namely an averaged water solubility at 25 °C of around 30%, the *Type 1* and *Type 2* bio-films show an improvement in terms of water solubility resistance by confirming, instead, the water solubility of the bio-films constituted by just powder and plasticizer.

## 4. Conclusions

This work proposed an innovative method for producing biodegradable thermoplastic polymeric films by employing waste flours derived from pasta factory (DWF) and pizzeria (SWF) waste. The granulometric and diffractometric analyses evidenced a difference between the neat flours and the waste ones due to the several working processes that the waste powders had previously undergone, changing their structures in terms of particle size and crystallinity. The optimal amounts of glycerol able to plasticize the waste flours, i.e., 60% and 80% for DWF and SWF, respectively, have been determined through the realization of different blends through employing a controlled method with the subsequent steps of mixing, fusion and compression molding. From the mechanical tests it was determined that *Type 0* bio-films showed low mechanical properties. For this reason, PLA and PLA plasticized with cardanol were added in order to produce *Type 1* and *Type 2* bio-films, respectively, reaching tensile properties comparable to those reported in the literature. Furthermore, both *Type 1* and *Type 2* waste-based bio-films showed a reduction in the moisture absorption by about 60% and reductions in the water vapor permeability by 35% to 50% with respect to the *Type 0* blends. Artificial ageing affected *Type 0* and *Type 2* bio-films when exposed to UV radiation by causing an embrittling due to the loss of the plasticizers (glycerol and cardanol); on the other hand, the condensation exposure affected the *Type 1* bio-films more, in which the lower content of plasticizer was compensated by the water, thus increasing the ductility of the bio-films. Moreover, PLA was also affected from the aesthetical point of view, inducing an evident bleaching in *Type 1* and *Type 2* bio-films. Finally, the *Type 1* and *Type 2* bio-films showed an averaged biodegradation at 50 °C of around 30% to 40% with respect to maize-starch-based bio-films, which totally dissolved within 24 h. In light of the obtained results, the waste flours could be proposed as suitable substitutes for neat maize starch to produce biodegradable thermoplastic polymeric films. This is an important goal to achieve in order to avoid the agricultural exploitation of the production of neat starch for industrial applications by promoting, on the contrary, the production of biodegradable thermoplastic polymers derived from the recycling of organic waste matter.

## Figures and Tables

**Table 1:** Composition of the blends: maize starch (MS), glycerol (G), durum waste flour (DWF), soft waste flour (SWF), polylactic acid (PLA) and cardanol (C) contents.

Blend *Type*	Blend Code	MS (wt %)	DWF (wt %)	SWF (wt %)	Glycerol (wt %)	PLA (wt %)	Cardanol (wt %)
*Type 0*	MS50G	66.67	0	0	33.33	0	0
	MS60G	62.50	0	0	37.50	0	0
	MS70G	58.83	0	0	41.17	0	0
	DWF50G	0	66.67	0	33.33	0	0
	DWF60G	0	62.50	0	37.50	0	0
	DWF70G	0	58.83	0	41.17	0	0
	SWF60G	0	0	62.50	37.50	0	0
	SWF70G	0	0	58.83	41.17	0	0
	SWF80G	0	0	55.56	44.44	0	0
*Type 1*	MS60G_PLA (50/50)	31.25	0	0	18.75	50	0
	DWF60G_PLA (80/20)	0	50	0	30	20	0
	DWF60G_PLA (70/30)	0	43.75	0	26.25	30	0
	DWF60G_PLA (50/50)	0	31.25	0	18.75	50	0
	SWF60G_PLA (80/20)	0	0	50	30	20	0
	SWF60G_PLA (70/30)	0	0	43.75	26.25	30	0
	SWF60G_PLA (50/50)	0	0	31.25	18.75	50	0
*Type 2*	MS60G_PLA30C (50/50)	31.25	0	0	18.75	35	15
	DWF60G_PLA30C (50/50)	0	31.25	0	18.75	35	15
	SWF80G_PLA30C (50/50)	0	0	27.78	22.22	35	15

**Table 2:** Tensile properties of the maize starch (MS)-based, durum waste flour (DWF)-based and soft waste flour (SWF)-based bio-films.

Blend *Type*	Bio-Film	σ_R_ (*MPa*)	ε_R_ (%)	*E* (*MPa*)
*Type 0*	MS60G	0.20 ± 0.06	91 ± 5	1.71 ± 0.72
	DWF60G	0.16 ± 0.04	23 ± 5	1.38 ± 0.26
	SWF80G	0.11 ± 0.04	22 ± 7	0.84 ± 0.3
*Type 1*	MS60G_PLA (50/50)	14.08 ± 3.71	5 ± 1.7	851.99 ± 216.72
	DWF60G_PLA (80/20)	2.63 ± 0.56	8.9 ± 3.2	86.57 ± 24.67
	DWF60G_PLA (70/30)	4.10 ± 0.86	11.2 ± 3.3	126.88 ± 24.11
	DWF60G_PLA (50/50)	10.5 ± 0.97	5 ± 1	719.2 ± 83.01
	SWF80G_PLA (80/20)	1.13 ± 0.19	5.58 ± 2.28	55.8 ± 39.24
	SWF80G_PLA (70/30)	3.66 ± 0.55	7.51 ± 1.97	183 ± 19
	SWF80G_PLA (50/50)	7.53 ± 1.14	5.06 ± 0.95	236.64 ± 52.54
*Type 2*	MS60G_PLA30C (50/50)	1.64 ± 0.2	231 ± 25	5.03 ± 2.89
	DWF60G_PLA30C (50/50)	1.85 ± 0.23	168 ± 63	10.55 ± 5.91
	SWF80G_PLA30C (50/50)	0.39 ± 0.17	99 ± 32	4 ± 3.75

**Table 3:** Tg values of the maize starch (MS)-based, durum waste flour (DWF)-based and soft waste flour (SWF)-based bio-films as determined by dynamic mechanical analysis (DMA) and by differential scanning calorimetry (DSC).

Blend *Type*	Bio-Film Type	Tg (°C) by DMA	Tg (°C) by DSC
*Type 0*	MS60G	70.6	71.5
	DWF60G	78.7	80.3
	SWF80G	80.1	82.3
*Type 1*	MS60G_PLA	51.3	53.5
	DWF60G_PLA	60.1	62.4
	SWF80G_PLA	60.5	62.9
*Type 2*	MS60G_PLA30C	47.8	49.7
	DWF60G_PLA30C	52.6	54.2
	SWF80G_PLA30C	54.3	56.5

**Table 4:** Hygroscopic and water vapor permeability data of maize starch (MS)-based, durum waste flour (DWF)-based and soft waste flour (SWF)-based produced bio-films.

Blend *Type*	Bio-Film	Imbibition Coefficient, α (%) at 23 °C, 75% RH	Slope (g/h)	Water Vapor Permeability (g/m^2^ Day) at 23 °C, 90% RH	Water Vapor Transmission Rate ((g/m^2^ day)/s)
*Type 0*	MS60G	12.84	0.06	970.49	0.14
	DWF60G	23.84	0.32	921.10	0.11
	SWF80G	24.68	0.38	839.54	0.06
*Type 1*	MS60G_PLA	5.53	0.01	211.91	0.02
	DWF60G_PLA	9.44	0.09	486.13	0.06
	SWF80G_PLA	10.85	0.12	547.89	0.05
*Type 2*	MS60G_PLA30C	7.94	0.02	291.98	0.02
	DWF60G_PLA30C	10.55	0.08	555.28	0.07
	SWF80G_PLA30C	10.30	0.10	498.54	0.07

**Table 5:** Color data of maize starch (MS)-based, durum waste flour (DWF)-based and soft waste flour (SWF)-based bio-films before and after QUV ageing.

		Before QUV Ageing			After QUV Ageing			Color Difference			
Blend *Type*	Bio-Film	L*	a*	b*	L*	a*	b*	*Δ* L*	Δa*	Δb*	ΔE*
*Type 0*	MS60G	81.28	1.65	25.37	82.79	0.56	21.47	1.51	−1.09	−3.90	4.32
	DWF60G	18.48	24.00	26.73	15.89	22.19	19.78	−2.59	−1.81	−6.95	7.64
	SWF80G	54.21	33.69	66.32	64.51	30.27	69.18	10.29	−3.42	2.86	11.22
*Type 1*	MS60G_PLA	61.07	29.71	56.44	34.00	5.89	11.83	−27.08	−23.82	−44.61	57.37
	DWF60G_PLA	9.11	19.93	8.54	52.79	2.52	6.13	43.68	−17.41	−2.41	47.08
	SWF80G_PLA	20.79	40.91	32.97	18.66	6.17	11.41	−2.14	−34.74	−21.57	40.94
*Type 2*	MS60G_PLA30C	23.81	41.87	33.77	36.92	31.36	33.68	13.10	−10.52	−0.09	16.80
	DWF60G_PLA30C	12.78	27.09	17.89	31.13	7.21	12.21	18.35	−19.88	−5.68	27.64
	SWF80G_PLA30C	12.97	12.49	13.22	33.18	5.29	10.85	20.21	−7.20	−2.37	21.58

## Supplementary Materials

The “Supporting Figures and Tables” file, referred to Granulometric, Diffractometric and Calorimetric analyses of the flours, is available online at https://www.mdpi.com/2073-4360/12/6/1414/s1.
