# Supplementary material for: An Innovative Method for the Recycling of Waste Carbohydrate-Based Flours
Source: Polymers (Basel). 2020 Jun 24;12(6):1414. doi: 10.3390/polym12061414 (PMC7362212; doi:10.3390/polym12061414)
Supplement: Supplementary file 1 [file polymers-12-01414-s001.pdf]

Supporting Figures and Tables

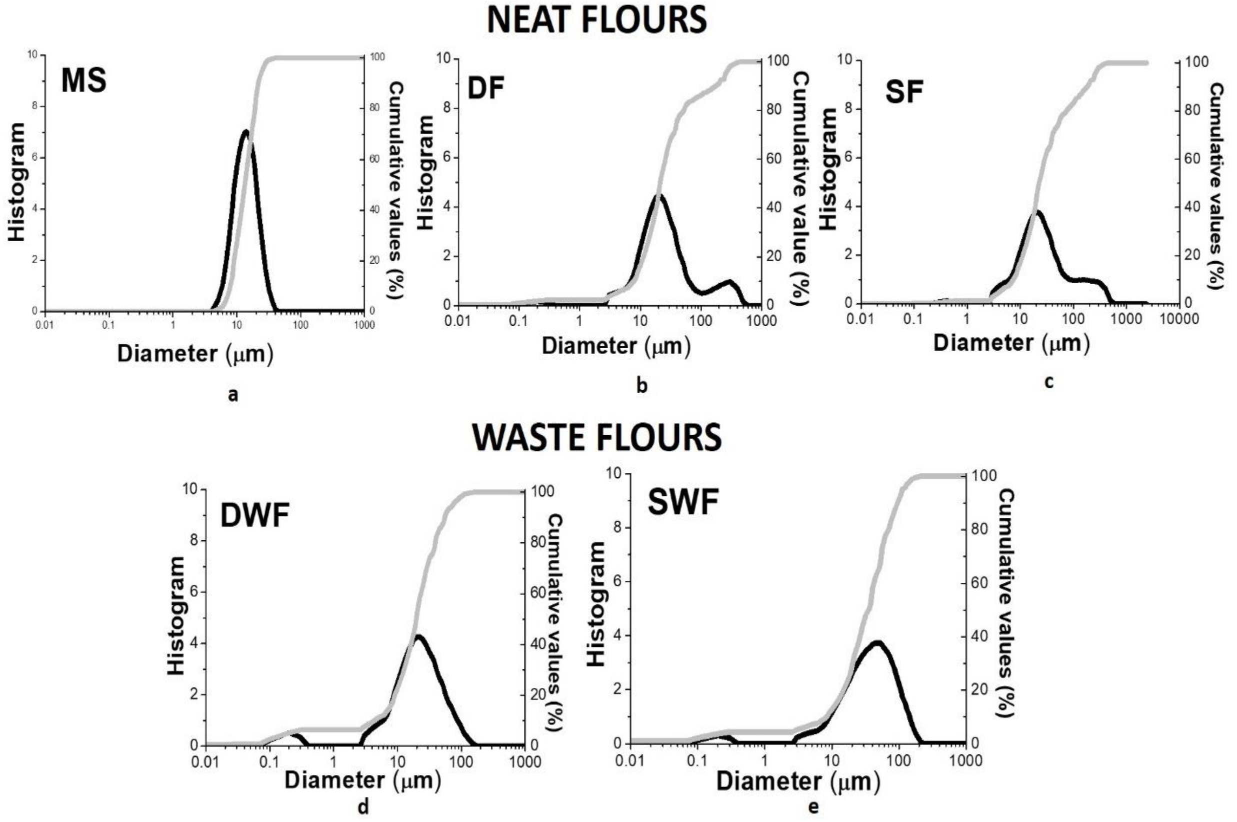

Figure S1. Granulometric distributions of neat powders: MS (a), DF (b) and SF (c), compared to the waste flours: DWF (d) and SWF (e).

Table S1. Particles diameter of neat (MS, DF, SF) and waste (DWF, SWF) flours at different cumulative values (CV).

CV (%)	PARTICLES DIAMETER (μm)				
	NEAT FLOURS			WASTE FLOURS	
	**MS**	**DF**	**SF**	**DWF**	**SWF**
**10%**	8.38	8.23	7.82	5.30	8.76
**50%**	14.32	23.6	25.44	21.23	37.61
**90%**	24.45	184.82	188.83	60.10	99.83

By observing the histograms (black curves) reported in Figure S1-b and Figure S1-c, both, durum and soft neat flours, respectively, show a similar double distribution, unlike the MS (Figure S1-a) that shows a homogeneous distribution centred around 10 μm. The granulometric analysis of the waste flours reported in Figure S1-d and Figure S1-e evidenced two peaks, but centred at different diameter values with respect to the as produced flours. In fact, on one side, the particles' sizes of DF and SF are very comparable to each other by recording cumulative values around 8μm (for CV equal to 10%), 23 μm (for CV equal to 50%) and 185 μm (for CV equal to 90%), as reported in Table S2. On the other hand, for both the waste flours, the first peak is centred around 0.2 μm, corresponding to the 2.5% of the total distribution, and the second one is centred at 21.23 μm for DWF and 37.61 μm for SWF, corresponding to the 50% of the distribution.

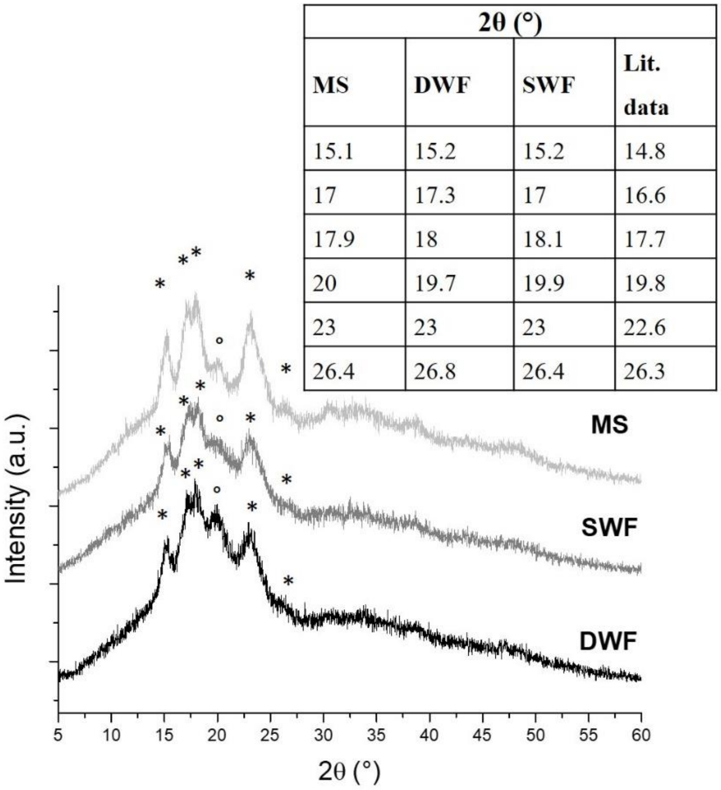

2θ (°)			
**MS**	**DWF**	**SWF**	**Lit. data**
15.1	15.2	15.2	14.8
17	17.3	17	16.6
17.9	18	18.1	17.7
20	19.7	19.9	19.8
23	23	23	22.6
26.4	26.8	26.4	26.3

Figure S2. XRD spectra of MS, DWF and SWF powders and the comparison of characteristic peaks of the powders with those reported in the literature [1] in the inset.

Table S2. Crystallinity data derived from the XRD analysis

SAMPLE	XRD Relative Cristallinity (%)
**MS**	54.05
**DF**	4.77
**SF**	10.51
**DWF**	26.27
**SWF**	11.59

For each spectrum, it is possible to note the presence of the peaks at around 15°, 18°, and 23°, that correspond to those expected from the A-type crystalline structures, the typical crystalline structure of cereal starches, as widely studied by [2]. The B-type crystalline structure, instead, is proper for the tuber, fruit and stem starches, such as potato and banana starches. Wheat starch contains two kinds of starch granules, the large disk-shaped A-type ones and the small spherical and irregular B-type ones [3–5]. Both, the A- and B-type granules are predominantly present in the A-type crystalline structure of wheat starch, by conferring to the latter several properties as function of the ratio of A/B-type granules. Kim and Huber [6] studied as the A/B-type granules ratio influenced the swelling, gelatinization, and pasting properties of wheat starch, due to the different distribution of the amylopectin chain lengths between the two types of granules. By analysing the spectra reported in Figure S, the main diffraction peaks positioned at 15°, 17°, 18° and 23° correspond to the typical A-type crystalline structure of both A- and B-type granules; the peak at around 20° of the B-type granules is characterized by a V-type crystalline structure, that describes the amylose single helices co-crystallized with some compounds, as reported by [7].

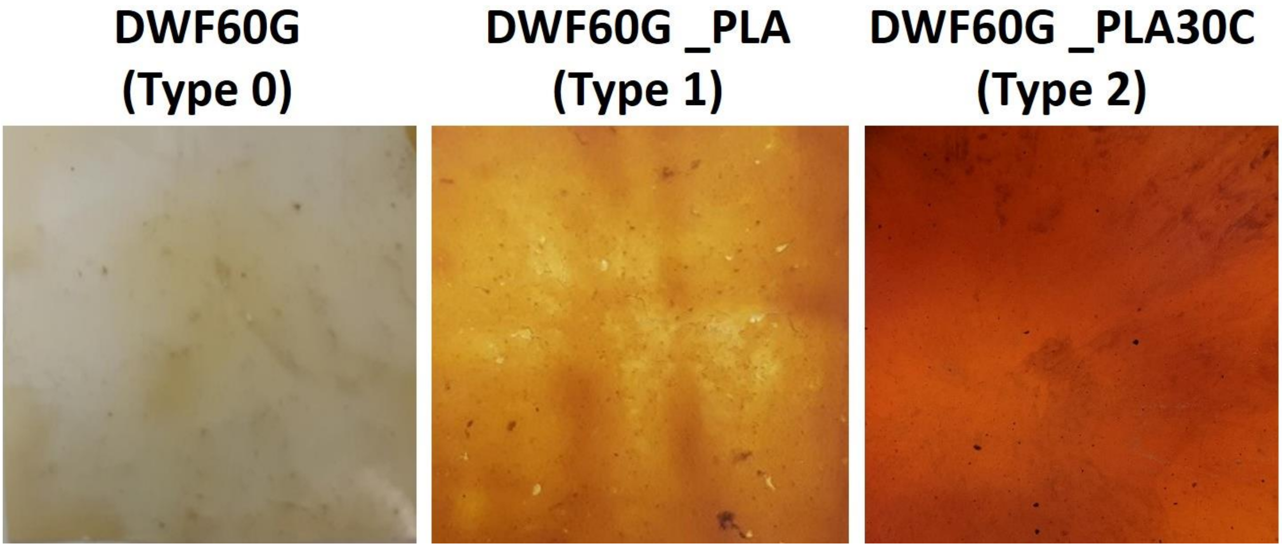

Figure S2. *Type* 0, *Type* 1 and *Type* 2 examples of realized bio-films

Figure S4 shows DSC thermograms of (a) MS, (b) DWF and (c) SWF samples. The glass transition values, calculated as the inflection point of the curves, are comparable to those obtained with DMA analysis. In particular, for all samples, a lowering of Tg was found with the addition of PLA, which is characterized by a glass transition of about 60 °C. The further addition of cardanol involved a higher decrease of the Tg, due to its plasticizing effect. Nevertheless, a progressive increase in glass transition was detected moving from *Type* 0 to *Type* 2 system.

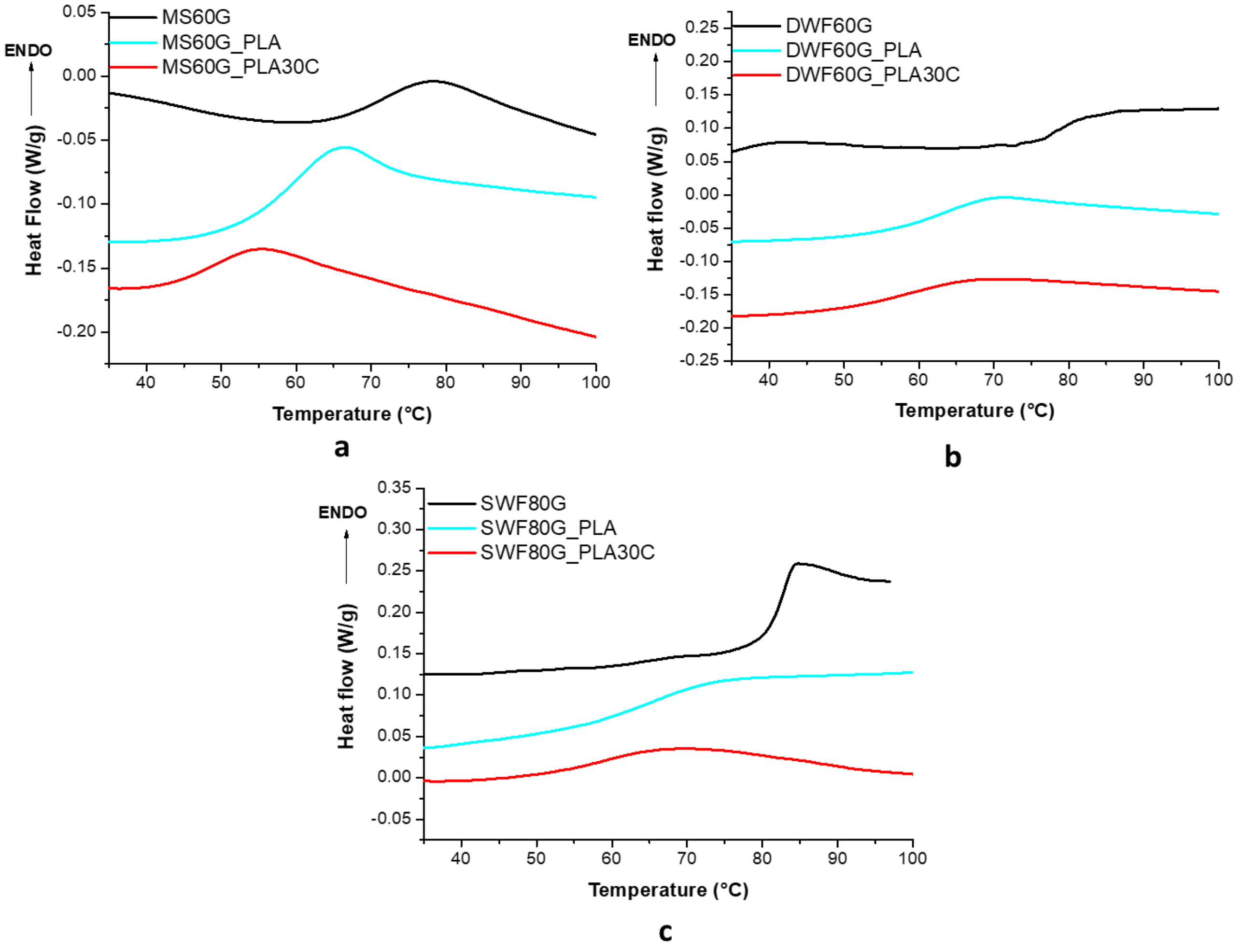

Figure S4. DSC curves of (a) MS, (b) DWF and (c) SWF samples.

Supporting References

1. Van Soest, J.; Hulleman, S.; DeWit, D.; Vliegenthart, J. Crystallinity in Starch bioplastics. *Ind Crop. Prod.* **1996**, *56*, 11–22.

2. Zhang, B.; Li, X.; Liu, J.; Xie, F.; Chen, L. Supramolecular structure of A- and B-type granules of wheat starch. *Food Hydrocoll.* **2013**, *31*, 68–73.

3. Wei, C.; Zhang, J.; Chen, Y.; Zhou, W.; Xu, B.; Wang, Y.; Chen, J. Physicochemical properties and development of wheat large and small starch granules during endosperm development. *Acta Physiol. Plant.* **2010**, *32*, 905–916.

4. Hayashi, M.; Kiribuchi-Otobeb, C.; Seguchi, M. Ghosts of b-type wheat starch granules in concentrated KI/I2 solution. *Starch/Stärke* **2005**, *57*, 384–387.

5. Zihua Ao; Jane, J. Characterization and modeling of the A-and B-granule starches of wheat, triticale, and barley. *Carbohydr. Polym.* **2007**, *67*, 46–55.

6. Kim, K.S.; Huber, K.C. Physicochemical properties and amylopectin fine structures of A- and B-type granules of waxy and normal soft wheat starch. *J. Cereal Sci.* **2010**, *51*, 26–264.

7. Buleon, A.; Colonna, P.; Planchot, V.; Ball, S. Starch granules: structure and biosynthesis. *Int. J. Biol. Macromol.* **1998**, *23*, 85–112.
